# Establishment of a nomogram-based prognostic model (LASSO-Cox regression) for predicting platelet storage lesions under different storage conditions

**DOI:** 10.3389/fmolb.2025.1561114

**Published:** 2025-03-31

**Authors:** Jun Xiao, Huimin Li, Xiaowei Li, Huifen Lei, Zhicai Li, Cuiying Li

**Affiliations:** ^1^ Department of Blood Transfusion, Air Force Medical Center, Air force medical University, Beijing, China; ^2^ The Fifth School of Clinical Medicine, Anhui Medical University, Hefei, China

**Keywords:** platelet concentrates (PCs), platelet storage lesions (PSLs), miRNA sequencing, Lasso-Cox regression, nomogram

## Abstract

**Introduction:**

Platelet concentrates (PCs) are critical blood products used for transfusion, but stored platelets often experience quality deterioration, resulting in reduced efficacy post-transfusion. Currently, the lack of effective prediction models hinders the assessment of platelet storage quality. To address this, we developed a miRNA-based prognosis prediction model that comprehensively evaluates platelet quality under diverse storage conditions, offering valuable insights into platelet shelf life.

**Methods:**

We enrolled 249 eligible PC samples, divided into a training dataset and internal validation dataset (7:3). Through microRNA sequencing, we identified 13 differentially expressed miRNAs with platelets storage lesions (PSLs). Leveraging the LASSO-Cox regression model, we constructed a nomogram-based classifier based on the association between miRNA expression and the duration of PSLs-free survival. Performance evaluation using measures like concordance index, area under the curve, calibration curves, and decision curve analyses to confirm the model’s robustness.

**Results:**

The nomogram classifier, incorporating miRNAs (miR-4485-3p, miR-12136, miR-25-5p, miR-148b-5p) and storage method, effectively categorized PCs into high-risk and low-risk groups. Notably, significant differences in PSLs-free survival were observed across all datasets, underscoring the precision and accuracy of our nomogram-based model.

**Discussion:**

This innovative classifier provides clinicians with a reliable tool to predict PSLs occurrence in PCs stored under different methods, facilitating improved clinical decision-making.

## 1 Introduction

Platelet concentrate (PC) transfusion is a life-saving strategy to control and prevent bleeding in hematological, trauma, surgical and cancer patients (Ng et al., 2018), and plays major roles in hemostasis, inflammation and wound healing (Jurk and Kehrel, 2005). However, the storage of platelets is associated with several challenges, including the development of storage lesions that can compromise their functionality and lead to adverse reactions in recipients. Alternatively, cold stored (2°C–6°C) PC have decreased bacterial contamination risks and extended the shelf life compared to room temperature PC (Nair et al., 2014). However, posttransfusion platelet survival was reduced due to cold-induced platelet activation (Brown et al., 2022; Egidi et al., 2010), resulting in an increased the occurrence of PSLs. Currently, PSLs occur due to platelet metabolism under *in vitro* preparation and storage pressures. These changes ultimately lead to disturbances in platelet activation, aggregation, secretion and immune functions (Ng et al., 2018). Platelet storage lesions manifest differently depending on the specific storage conditions and duration. For example, at 22°C, the extent of platelet storage lesions increases gradually with prolonged storage time (Rajashekaraiah and Rajanand, 2023). Regarding platelets stored at low temperature (4°C), bacterial contamination is minimized and storage duration is prolonged under these conditions (Liu et al., 2024). However, the substantial stress caused by low temperature may result in irreversible lesions, which lead to rapid platelet clearance (Jimenez-Marco et al., 2022). Furthermore, the accuracy of *in vitro* tests in predicting the *in vivo* recovery and survival of transfused platelets is not satisfactory (Shrivastava, 2009). Additionally, using any of these risk factors cannot fully reflect the degree of PSLs, while the combined use of these risk factors will lead to an increase in medical costs. Therefore, the development of a reliable prognostic model to predict the occurrence and severity of platelet storage lesions under different storage conditions is a significant step forward in enhancing patient care and ensuring the optimal use of platelet transfusions.

microRNA (miRNA), approximately 21–23 nucleotides length, is a class of endogenous noncoding RNAs with regulatory functions. They play a role in controlling the expression of mRNA by primarily binding to mRNA in a complementary manner (Landry et al., 2009; Pontes et al., 2015). At present, a few miRNAs have been discovered to be expressed in platelets and to play a role in controlling the expression of genes within platelets. Previous research suggested that let-7b and miR-16 showed increasing levels during storage while miR-7 and miR-145 showed decreasing levels (Kannan et al., 2009), which indicated the use of miRNAs as biomarkers for stored platelet quality during different storage conditions (Maues et al., 2019; Norouzi et al., 2021). In comparison to using only one predictor, integrating multiple predictors into a single model would significantly increase prognostic value (Maues et al., 2018; Maues et al., 2020; Mi et al., 2022).

With the deep sequencing technology of biological microarrays, thousands of target genes or miRNAs can be evaluated simultaneously, and the number of covariates is equal to or more than the number of observations. When the sample size to covariate ratio is too low (such as less than 10:1), Cox proportional hazards regression analysis, the most popular method for modeling covariate information for survival times, does not work well for high-dimensional data (Simon and Altman, 1994). To overcome this restriction, the least absolute shrinkage and selection operator approach (LASSO) was developed (Tibshirani, 1997). Although miRNAs have been proven to be associated with PSLs, they are scarcely applied to the measurement of PC prognoses under different storage environments.

In this study, we developed a multi miRNA-based classifier with the LASSO-Cox regression model to predict PSLs-free duration based on the training dataset. The prognostic and predictive accuracy of this classifier were assessed in internal and external datasets. Finally, by classifying the platelets into low- and high-risk strata, we were able to confirm the potential use of our nomogram in predicting PSLs-free duration.

## 2 Materials and methods

### 2.1 Study design and sample selection

**FIGURE 1:**
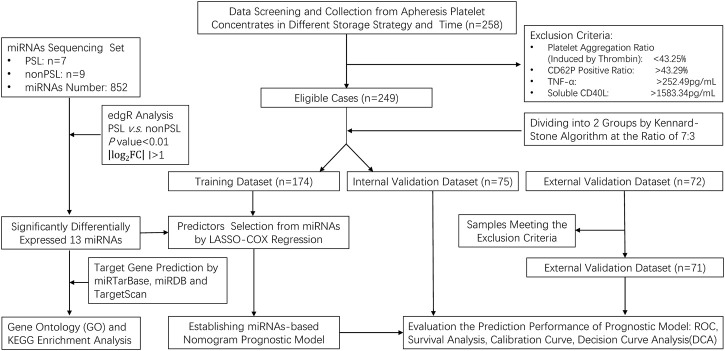
Flow chart of the study design and analysis strategy.

We performed a prospective study of PC under different storage strategies. In this investigation, a PC was seen as a single case, and 258 cases matching preset inclusion and exclusion criteria were enrolled. Platelets were collected by apheresis (FRESENIUS KABI, Germany) and stored in the platelet storage bags (S5L Platelet Set, FRESENIUS KABI, Germany) with natural plasma. All PC was stored at 22°C ± 2°C or 4°C ± 2°C with constant agitation on a platelet shaker for up to 10 days. To simulate the application scenario of prolonged and long-distance transportation, we incorporated an experimental study on the storage of platelets at room temperature within a blood transport box (HZY-15Z, Haier, Qingdao, China), maintaining continuous agitation for a duration of 7 days. The complete data were applied from the abovementioned samples to establish a nomogram-based prognostic model, which involved seven steps in the study procedures (Figure 1).

First, we enrolled 249 platelet samples from healthy blood donors, who were qualified to donate blood, and selected eligible cases with our inclusion and exclusion criteria based on the aggregation ratio and the expression of CD62P positive ratio, TNF-α and soluble CD40L (sCD40L) platelets. These parameters were assessed throughout the storage period to capture the dynamic changes that occur during the storage of platelets. Platelet aggregation was assessed using a platelet aggregometer (Chrono-log, United States). Platelet aggregation was induced by the addition of thrombin (1U/L). The aggregation was monitored in real-time and expressed as the percentage of aggregation relative to the initial platelet count. CD62P expression was evaluated using flow cytometry. Briefly, platelets were stained with fluorescent-conjugated monoclonal antibodies specific for CD62P, and the expression was analyzed using a BD FACS flow cytometer. The data were processed using BD FACS software. TNF-α and sCD40L levels in the plasma were measured using an enzyme-linked immunosorbent assay (ELISA) kit from Shanghai Enzyme-linked Biotechnology (Shanghai, China), following the manufacturer’s instructions. The absorbance was read at 450 nm using Microplate Reader M5 (Molecular Devices, United States), and the concentrations were determined by comparing the optical density of the samples to the standard curve.

After collecting the complete data from the PC, we calculated the reference range based on the mean and standard deviation of each factor. Samples below the lower limit of the reference range of the aggregation ratio (<43.25%), or above one of the upper limits of the reference range of the CD62P positive ratio (>43.29%), TNF-α (>252.49 pg/mL) or sCD40L (>1583.34 pg/mL) will be excluded from the cohort of this study. Second, the 249 cases were divided into training dataset (n = 174) and an internal validation dataset (n = 75) by the Kennard–Stone algorithm (7:3), to ensure enough samples for constructing the prediction model. Third, 13 miRNAs were selected from the miRNA sequencing that were most significantly expressed (|log2 (fold change) |> 1.0, P < 0.01) in the PSLs cohort. Fourth, a certain number of prognostic miRNAs were screened by using least absolute shrinkage and selection operator (LASSO)-Cox regression model analysis. Fifth, a prognostic model was constructed and presented as a prognostic nomogram. Sixth, the prognostic prediction model was validated and adjusted based on internal and external validation datasets (n = 71), and the prediction performance was detected by: discrimination (receiver operating characteristic curve, ROC), calibration, and decision curve analysis (DCA). Finally, Kaplan–Meier curves and the log-rank test were further used to examine the discrimination of all eligible cases by risk scores.

### 2.2 Inclusion and exclusion criteria

All platelet concentrates from a single donor contained more than 2.5 × 10^11^/unit, white blood cells were less than 5 × 10^8^/unit, and red blood cells were less than 8 × 10^9^/unit (Mi et al., 2022). The exclusion criteria were described above.

### 2.3 Outcomes measurement and variable definition

The endpoint was set as the occurrence of PSLs, and the follow-up time was defined as the period from the date of the first day of platelet preparation to the date of PSLs or the date of last follow-up. The criteria for defining PSLs in this study were that the aggregation ratio of the platelet sample must be below the lower limit of the reference range (<43.25%) and meet one of the following conditions: above one of the upper limits of the reference range of the CD62P positive ratio (>43.29%), TNF-α (>252.49 pg/mL) or sCD40L (>1583.34 pg/mL). When the sample meets any of the above conditions, it will be processed as censored data during the follow-up period.

### 2.4 Analysis of miRNA profile by high throughput sequencing

Samples from 7 PSLs and 9 non-PSLs were employed to conduct the miRNA sequencing analysis. Apheresis platelets were used to collect single-donor platelets, then centrifuged them to get platelet concentrate (PC) with about 1 mL of plasma. The PC was centrifuged again, washed with saline, and centrifuged once more. The resulting platelet pellet was resuspended in 0.1 mL of saline for miRNA extraction. Total RNA, including miRNAs, from 0.1 mL PC obtained from the above procedures was extracted by a miRNeasy Serum Plasma kit (Qiagen, Germany) following the manufacturer’s instructions. RNA purity and integrity were analyzed by a NanoPhotometer® spectrophotometer (IMPLEN, CA, United States) and a Bioanalyzer 2100 with an Agilent RNA 6000 Nano Kit (Agilent Technologies, CA, United States) respectively. Small RNA sequencing libraries were constructed using the NEBNext Multiplex Small RNA Library Prep for Illumina (New England Biolabs, MA, United States). Next, small RNAs were sequenced on an Illumina Hiseq 2500/2000 platform. The raw sequencing data have been deposited in the National Center for Biotechnology Information’s Gene Expression Omnibus (GSE243571).

Differential expression analysis of PSLs/non-PSLs was performed using the edgeR R package. A *P* value < 0.01 and |log_2_ (fold change)| > 1.0 were set as the thresholds for significant differential expression by default.

TargetScan, miRDB, and miRTarBase were used to predict the target genes of differential miRNAs. To further elucidate the biological function of these target genes, we performed Gene Ontology (GO) biological enrichment analysis and Kyoto Encyclopedia of Genes and Genomes (KEGG) pathway analysis (www.kegg.jp/kegg/kegg1.html) (Kanehisa and Goto, 2000) through the DAVID bioinformatics database (https://david.ncifcrf.gov/). A significance level of false discovery rate (FDR) of less than 0.05 was set as the cutoff criteria.

### 2.5 Differential miRNA expression by quantitative real-time polymerase chain reaction (qRT-PCR)

Based on the miRNA sequencing results, miRNA expression was analyzed by using qRT-PCR to analyze the 320 platelet samples in different datasets to assess and validate the prognostic value of each differential miRNA. Reverse transcription reaction and qRT-PCR were performed in the miDETECTA TrackTM miRNA qRT-PCR Starter Kit (RiboBio, Guangzhou, China) on the ABI7500 Real-Time PCR System (ABI) following the manufacturer’s instructions. The RNU6 gene was selected as the internal reference gene and the 2^−△△Ct^ method was used to analyze the data (Norouzi et al., 2021).

### 2.6 Preliminary data processing

For data processing, the miRNA expression data, recorded as continuous variable, were converted into binary variables by using X-tile software version 3.6.1 (Yale University School of Medicine, CT, United States). The X-tile selected the optimum cutoff value for the expression of every miRNA based on the association with the PCs samples’ PSLs-free survival. X-tile plots offer a convenient and straightforward approach for evaluating the link between variables and survival outcomes. With the assistance of X-tile software, researchers can automatically determine the most suitable data cutoff point based on the highest χ^2^ value (smallest p value) obtained from Kaplan-Meier survival analysis and the log-rank test (Supplementary Table S1).

### 2.7 Statistical analysis

R software (v4.4.2, http://www.Rproject.org) and MedCalc software version 22.009 (Ostend, Belgium) were applied to the statistical analysis in the present study. The normality test was performed by the Kolmogorov–Smirnov method. The original data are described as the median (interquartile range, IQR). The LASSO-Cox regression model was performed with the “glmnet” package, and the nomogram and calibration were conducted with the “rms” package. The “survivalROC” and “stdca.R” package were utilized to perform the ROC curve and DCA curve analysis, respectively. The Kaplan-Meier (K-M) method, conducted with the “survminer” package, was used to analyze the correlation between variables and PSLs-free duration, and the log-rank test was used to compare the survival rates.

Univariate analysis was conducted by the Cox regression test to obtain hazard ratio (HR) and 95% confidence interval (CI). A LASSO-Cox regression model was used to select the most useful prognostic predictors of all the PSLs-associated variables identified with the training dataset, and constructed a multimiRNAs-based nomogram model was constructed for predicting the 3-, 5-, and 7-days PSLs-free duration of platelets with different storage strategies.

The concordance index (C-index) and calibration curve were generated to evaluate the performance of the nomogram. Additionally, validation tests performed based on the internal and external validation datasets were conducted by ROC curve analysis and calibration curves at 3-, 5-, and 7-days to evaluate the effectiveness of the nomogram. The ROC curve for the nomogram was generated based on the area under the curve (AUC). The 45-degree line in the calibration curve represents the performance of an ideal nomogram, in which the predicted outcome perfectly corresponds with the actual outcome (Zhang et al., 2013). Meanwhile, DCA was utilized to assess the net benefit of the nomogram in a platelet storage context. According to the cutoff risk score generated by X-tile in the training dataset, the samples in different datasets were divided into high- and low-risk score groups to evaluate the robustness of the model by K-M analysis. Statistical significance was set at 0.05.

## 3 Results

### 3.1 Sample baseline characteristics

The characteristics and PSLs-free duration of all three datasets, including the training dataset (n = 174), internal validation dataset (n = 75), and external validation dataset (n = 71), are summarized in Supplementary Table S3. The median follow-up time was 8.91 (IQR 8.66–9.15) days, and 92 (28.75%) of 320 samples developed PSLs during the follow-up period. To identify the PSLs, reference ranges were generated based on the mean and standard deviation of the aggregation ratio, CD62P positive ratio, TNF-α and sCD40L in the training dataset and internal validation dataset. The reference range for each marker was 43.25%–86.91% for the aggregation ratio, 25.03%–43.29% for the CD62P positive ratio, 158.13–252.49 pg/mL for TNF-α and 806.82–1583.34 pg/mL for sCD40L. The sample that met the exclusion criteria was excluded before the follow-up period. The complete data were applied from the abovementioned samples to perform the following analysis, which involved seven steps in the study procedures (Figure 1).

### 3.2 Bioinformatics analysis of platelet-derived miRNAs

**FIGURE 2:**
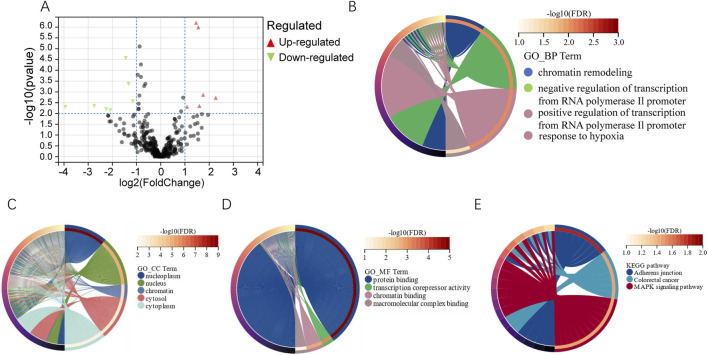
Differential analysis of miRNAs and functional analysis of their target genes. **(A)** Volcano map showing differential miRNAs expression. miRNA with *P* value < 0.01 and |log_2_FC| >1 was defined as differentially expressed genes. **(B–D)** GO analysis of biological processes, cellular components, and molecular functions for the target genes of differentially expressed miRNAs (*FDR* < 0.05). **(E)** Top 3 statistically significantly enriched pathways in KEGG analysis (*FDR* < 0.05, www.kegg.jp/kegg/kegg1.html).

miRNA sequencing detected 13 microRNAs expressed differently in the two groups (PSLs and non-PSLs). Of these, 6 microRNAs were upregulated and seven were downregulated (|log_2_ (fold change) |> 1.0, P < 0.01). The volcano plot of the 13 differentially expressed miRNAs between PSLs and non-PSLs is shown in Figure 2A. The target genes of the differential miRNAs were predicted by TargetScan, miRDB, and miRTarBase (Supplementary Table S2). Figures 2B–D shows the GO analysis. The target genes were mostly enriched in chromatin remodeling, negative regulation of transcription from the RNA polymerase II promoter, positive regulation of transcription from the RNA polymerase II promoter, and response to hypoxia (FDR<0.05, biological process); nucleoplasm, nucleus, chromatin, cytosol, and cytoplasm (FDR<0.05, cellular component); and protein binding, transcription corepressor activity, and chromatin binding (FDR < 0.05, molecular function). KEGG analysis showed that the target genes were mainly involved in adherents’ junctions, Colorectal cancer, and MAPK signaling pathway (FDR < 0.05, Figure 2E).

### 3.3 Predictors’ selection of the prognostic model

The 13 differentially expressed miRNAs were confirmed using qRT-PCR in the training and internal validation datasets. X-tile plot software was used to generate the optimum cutoff value of the differential miRNAs (Supplementary Table S1). When the miRNA expression level was lower than the cutoff, it indicated low expression status, while higher expression than the cutoff value indicated high expression status.

**FIGURE 3:**
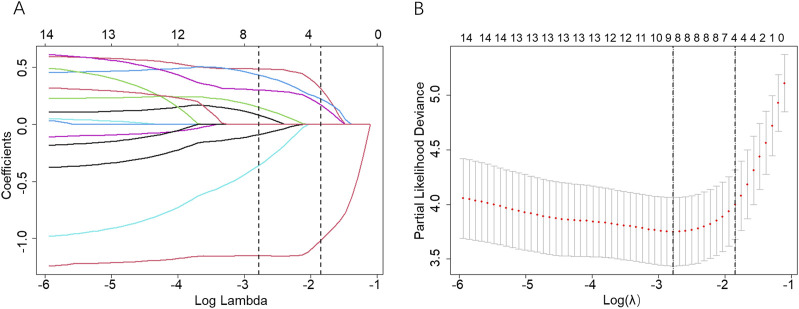
Screening of variables based on Lasso regression. **(A)** The variation characteristics of the coefficient of 14 variables; **(B)** The selection process of the optimum value of the parameter λ in the Lasso regression model by the cross-validation method. The two dashed vertical lines were sketched at the optimal λ value by minimum criteria and 1-s.e. criteria through 10-fold cross-validation.

The storage method and miRNA-associated predictors were found using LASSO-Cox regression for analyzing the training dataset (n = 174). First, 14 variables from the training dataset were analyzed by using univariable Cox regression (Supplementary Table S4), providing a guide for independent predictor selection. All the selected miRNAs were found to be statistically significant in the training dataset, which suggested that they might be implicated predictors for predicting PSLs-free duration. Second, a total of 14 selected variables (13 miRNAs and storage method) were entered into the LASSO model. As shown in Figure 3, the optimal log (lambda) and 1-s.e. log (lambda) were generated by 10-fold cross-validation based on minimum partial-likelihood deviance. We obtained 4 prognostic miRNAs: hsa-miR-4485-3p, hsa-miR-12136, hsa-miR-25-5p, and hsa-miR-148b-5p, and the predictive value of the selected 4 miRNAs was also evaluated using Kaplan-Meier survival analysis (Supplementary Figure S1). Third, the 4 miRNAs and storage method, as predictors for predicting PSLs-free duration, were analyzed using multivariate Cox regression (Table 2) which was put into a nomogram-based prognostic model.

### 3.4 Development of the nomogram-based prognostic model

**FIGURE 4:**
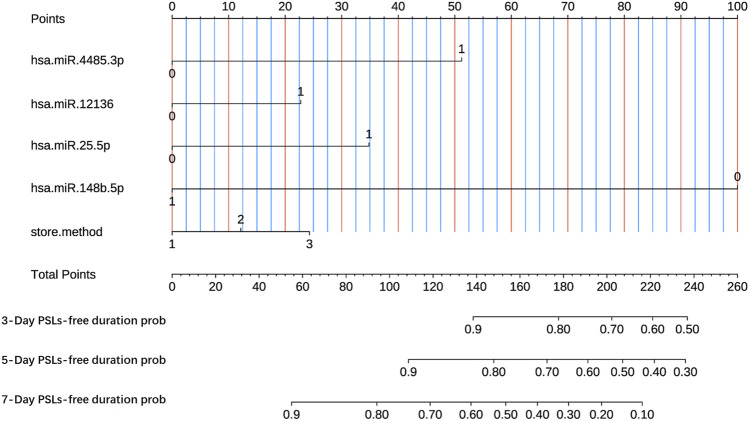
Nomogram-based prognostic model to predict 3-, 5- and 7-day PSLs-free duration of PCs under various storage strategies. The nomogram was established based on miR-4485-3p, miR-12136, miR-25-5p, miR-148b-5p, and the storage method. 3-Day survival prob, predicted probability of PSLs-free duration of 3 days; 5-Day survival prob, predicted probability of PSLs-free duration of 5 days; 7-Day survival prob, predicted probability of PSLs-free duration of 7 days.

Here, we developed a nomogram-based prognostic model based on the four-miRNA classifier according to the LASSO-Cox regression of the training dataset. Additionally, despite having no statistical significance in the training dataset, the storage method was also included in the prognostic model due to its clinical importance. As shown in Figure 4, the present nomogram-based prognostic model could graphically display predicted 3-, 5-, and 7-day PSLs-free duration. To illustrate the integrated correlation of the probability of PSLs-free duration with each predictor, a score was given to each subtype within each variable on a line segment with a scale of a certain percentage. In the end, the percentage transformed from a total accumulated score was used to quantify the accurately predicted PSLs-free duration probability. Based on the training dataset, the C-index was 0.881 (95% CI 0.840–0.922) in the nomogram model, while the C-index was 0.776 (95% CI 0.662–0.890) and 0.810 (95% CI 0.714–0.906) in the internal and external validation datasets, respectively.

### 3.5 Performance and validation of the nomogram

**FIGURE 5:**
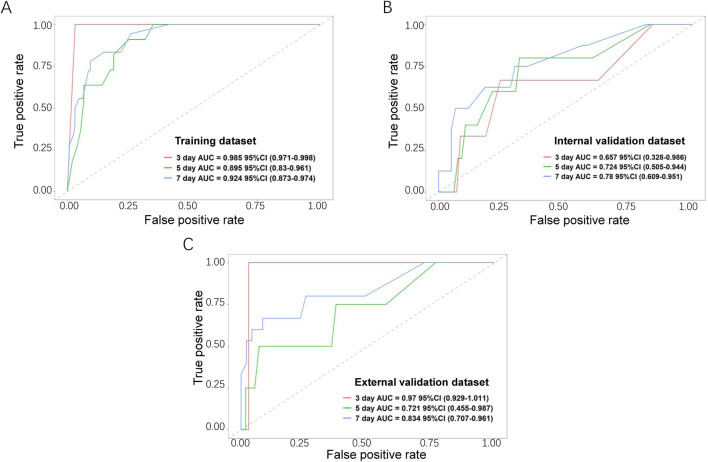
Receiver operating characteristic (ROC) curves for the nomogram-based model. Based on 3-, 5-, and 7-day PSLs-free duration, the ROC curves for the nomogram were plotted in the training dataset **(A)**, internal validation dataset **(B)**, and external validation dataset **(C)**. The red, green, and blue lines represent the curves for 3-, 5-, and 7-day ROC curves, respectively.

**FIGURE 6:**
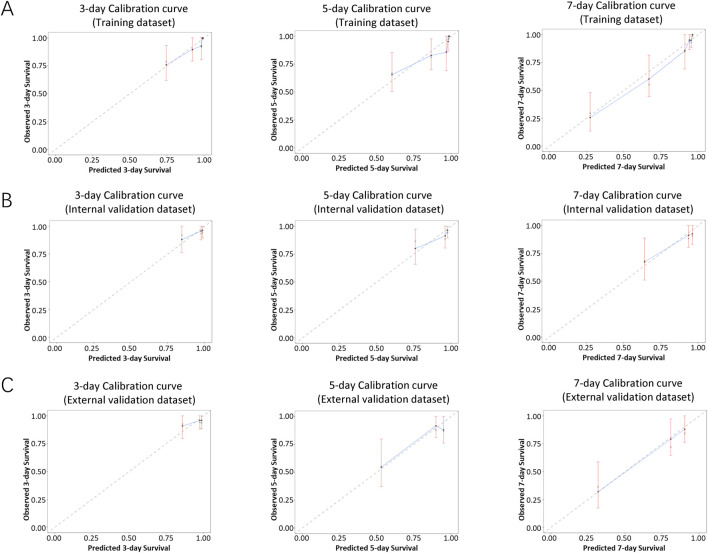
Calibration curves of the nomogram-based model. Calibration curves of the nomogram in the training dataset **(A)**, internal validation dataset **(B)**, and external validation dataset **(C)** were plotted based on 3-, 5-, and 7-day PSLs-free duration. The X-axis represents the model−predicted survival, while the Y−axis represents actual survival. The bars indicate 95% confidence intervals, and the dotted line represents the ideal reference line. The blue dots are calculated by bootstrapping (resample:500) and represent the nomogram performance. The closer the solid blue line aligns with the dotted line, the more accurate the model is in predicting PSLs-free duration.

**FIGURE 7:**
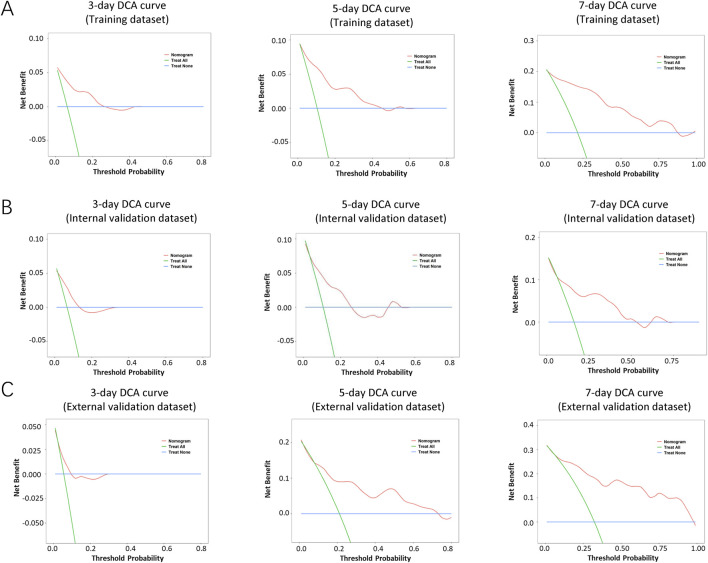
Decision curve analysis (DCA) of the nomogram-based model. The DCA curves of the nomogram in the training dataset **(A)**, internal validation dataset **(B)**, and external validation dataset **(C)** were plotted based on 3-, 5-, and 7-day PSLs-free duration, respectively. The net benefit calculated by adding true positives and subtracting false positive corresponds to the measurement of the Y-axis; the X-axis represents the threshold probability.

The performance of the nomogram was evaluated by time-dependent ROC curves, calibration curves and DCA. A time-dependent ROC curve was used to evaluate the false-positive rate and true positive rate of the model for the prognoses of PC. In the training dataset, the results showed that the areas under the curve (AUCs) of PSLs-free duration were 0.985, 0.895, and 0.924 at 3-, 5-, and 7-days, respectively (Figure 5A). In the internal validation dataset, the results showed that the areas under the curve (AUCs) of PSLs-free duration were 0.657, 0.724, and 0.780 at 3-, 5-, and 7-days, respectively (Figure 5B). In the external validation dataset, the results showed that the areas under the curve (AUCs) of PSLs-free duration were 0.985, 0.895, and 0.924 at 3-, 5-, and 7-days, respectively (Figure 5C). Calibration plots for 3-, 5-, and 7-days PSLs-free duration probabilities showed that the nomogram performed well compared with an ideal model in all three datasets (Figure 6). Consistently, the DCA curve also revealed good performance of the nomogram (Figure 7).

### 3.6 Application of risk stratification based on the nomogram model

Based on the nomogram model developed in the present study, we subdivided the samples into low-risk and high-risk groups (cutoff score: 112), and the risk stratification strategy showed good prognostic classification in the training dataset, internal and external validation datasets.

**FIGURE 8:**
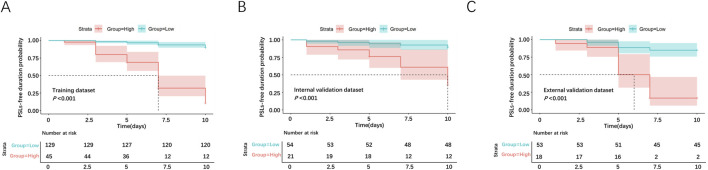
Survival analyses for PCs in the low-risk and high-risk groups. The survival curves were plotted in the training dataset **(A)**, internal validation dataset **(B)**, and external validation dataset **(C)**.

In the training dataset, there were 129 cases in the low-risk group and 45 cases in the high-risk group. The intergroup mean survival time was 9.72 (95% CI 9.51–9.93) days in the low-risk group and 6.89 (95% CI 6.10–7.68) days in the high-risk group, and the 5-day PSLs-free duration was 96.9% (95% CI 93.9–99.8) for the low-risk group and 68.9% (55.4–82.4) for the high-risk group (HR = 68.21, 95% CI 31.86–146.04, P < 0.001, Figure 8A).

In the internal validation dataset, there were 54 cases in the low-risk group and 21 cases in the high-risk group. The intergroup mean survival time was 9.56 (95%CI 9.06–10.05) days in the low-risk group and 7.88 (95%CI 6.51–9.24) days in the high-risk group, and 5-day PSLs-free duration was 94.4% (95%CI 88.3–100.5) for the low-risk group and 76.2% (58.0–94.4) for the high-risk group (HR = 14.43, 95%CI 4.83–43.12, P < 0.001, Figure 8B).

In the external validation dataset, there were 53 cases in the low-risk group and 18 cases in the high-risk group. The intergroup mean survival time was 9.25 (95% CI 8.74–9.75) days in the low-risk group and 6.17 (95% CI 5.11–7.23) days in the high-risk group, and 5-day PSLs-free duration was 88.7% (95% CI 80.2–97.2) for low-risk group and 50.0% (26.9–73.1) for the high-risk group (HR = 20.18, 95%CI 6.93–58.76, P < 0.001, Figure 8C).

## 4 Discussion

Despite the critical role of platelets in clinical medicine, their storage presents unique hurdles that can affect their viability and functionality. PSLs develop during PC preparation and storage (Chen et al., 2016; van der Meer and de Korte, 2011). During PC preparation, mechanical and biological forces cause platelet activation, which lasts the entire time of PC storage (Shukla et al., 2015; Maurer-Spurej et al., 2001). Platelet aggregation is hampered by alterations in platelet surface receptors and platelet-associated microparticle formation (Chen et al., 2016; Scott et al., 1983; Keuren et al., 2007). The homeostasis of inflammatory and immunomodulatory factors is disturbed by soluble factors and platelet-associated microparticles that accumulate during storage (Granai et al., 2023; Boncler et al., 2023; Morrell et al., 2014). In addition, PSLs have been therapeutically associated with decreased CCI and platelet survival (Ning et al., 2023; Spinella et al., 2019). Therefore, to reduce PSLs, changes in bag material and storage medium have been made recently to limit platelet glycolysis and activation (Lagerberg et al., 2015). Furthermore, cryopreservation and cold storage also have the ability to prolong storage, limit bacterial growth, and improve hemostasis (Nair et al., 2014; Wood et al., 2018; Ang et al., 2023). However, there are currently few methods available for identifying and predicting storage-specific PSLs to address storage-associated platelet activation and platelet survival reduction. In the present study, we examined markers associated with storage lesions, including platelet aggregation ratio, CD62P, sCD40L, and TNF-α. Utilizing data from both the training set and the internal validation datasets, we calculated the reference ranges for these markers to define whether platelets underwent storage lesions.

Advancements in omics technology, such as mass spectrometry proteomics, metabolomics and lipidomics; highly multiplexed affinity-based proteomics; microarray- or RNAsequencing-(RNA-seq)-based transcriptomics, and most recently ribosome footprint-based translatomics, are enhancing platelet detection, deepening our understanding of platelet biology (Gutmann et al., 2020). Despite this, these new methods face challenges in maturity, cost-effectiveness, and the expertise of test personnel, limiting their clinical use. Nucleic acid-based detection, however, can overcome these issues. Previous studies have reviewed numerous miRNAs that are differentially regulated during PC storage (Yan et al., 2017; Maues et al., 2018; Maues et al., 2019). Potential miRNAs revealed in the studies have been shown to work as effective biomarkers to pinpoint the molecular processes by which storage conditions for longer than 5 days cause storage-associated variations in PC (Maues et al., 2018; Mukai et al., 2019). In a study using miRNAs to predict platelet quality, the relative expression level of hsa-miR-127 was less than that of hsa-miR-320a, revealing that PC suffers aging *in vitro* and most likely exhibits storage lesions on platelets stored in a blood bank, suggesting that the PC bag is unsuitable for transfusion (Pontes et al., 2015). In addition, the upregulated miRNAs (miR-1304-3p, miR-432-5p, miR-411-5p, miR-668-3p, and miR-939-5p) were used to construct a specific strategic panel to conduct routine clinical trials of healthy blood bank donors by analyzing the differential expression of miRNAs (the fourth-day storage bags vs the first-day bags) (Maues et al., 2018). Additionally, elevated levels of miR-20a, miR-10a, miR-16–2, and miR-223 exhibited a positive correlation with platelet quality under cold (4°C) storage conditions.

Here, we employed small RNAs sequencing to reveal that 13 miRNAs exhibited differential expression between non-PSLs and PSLs, which were induced by distinct storage conditions (22°C, 4°C and room temperature with transportation). To clarify the differences between miRNA profiles obtained in previous studies and this study,we compared our RNA-Seq data with the miRNAs previously reported to be differentially regulated during PC storage. Additionally, a Venn diagram illustrating the overlap between our study and previous findings is provided in Supplementary Figure S2. The results indicate that only has-miR-25 was simultaneously identified in both our study and other literature. Furthermore, syntaxin binding protein 5 (STXBP5), identified as a potential target of hsa-miR-12136, has been linked to venous thromboembolism, a condition characterized by the formation of blood clots, secondary vascular changes, and altered hemodynamics (Zhu et al., 2014). This association implies that hsa-miR-12136 may influence platelet coagulation processes. Notably, platelets are implicated in the pathogenesis of myocardial infarction, stroke, and venous thromboembolism, while also playing a crucial role in mediating inflammation and the inflammatory response (Koupenova et al., 2018). Consequently, miRNAs derived from platelets may contribute to the regulation of inflammatory and immune responses. Empirical evidence has shown that miR-148b exerts a negative regulatory effect on dendritic cell (DC) maturation markers, such as HLA-DR and CD40, as well as on pro-inflammatory cytokines including TNF-α and IL-6. This regulatory action inhibits DC maturation and activation, thereby modulating the immune response (Conti et al., 2016). Additionally, Supplementary Table S2 reveals that MCM4, a putative target of miR-4485-3p, exhibits a significant correlation with immune cell infiltration. The recruitment of myeloid-derived suppressor cells (MDSCs) is promoted, while there is a negative correlation with natural killer T (NKT) cells (Li et al., 2024), suggesting that MCM4 may play a role in modulating the tumor microenvironment and mechanisms of immune evasion. Furthermore, these studies demonstrate that the differentially expressed miRNAs identified in this research exert a significant influence on platelet functions, potentially altering their behavior in both physiological and pathological contexts.

The target genes of the differential miRNAs in the present study were predicted by TargetScan, miRDB, and miRTarBase, and then GO and KEGG analysis was employed to assess the biological function. The findings revealed that the target genes were largely associated with chromatin regulation and the MAPK signaling pathway. Blocking this pathway has been found to result in superior maintenance the preservation of all platelet storage parameters *in vitro* (Skripchenko et al., 2013), as well as to significantly improve post-transfusion recovery (Canault et al., 2010). However, these investigations have yet to comprehensively appraise the predictive value of these differential miRNAs in the assessment of platelet quality under varying storage modalities. Moreover, they remain deficient in their evaluation of the prognostic implications of these miRNAs in the context of distinct platelet storage strategies.

In the present study, we employed a sequencing-based approach to screen differentially expressed miRNAs (hsa-miR-4485-3p, hsa-miR-12136, hsa-miR-25-5p, and hsa-miR-148b-5p; PSLs vs non-PSLs). Drawing upon these findings, we utilized the LASSO-Cox regression methodology to construct a miRNA-centric nomogram model, which provides a visual prognostic predictive framework. The advantage of LASSO regularization in feature selection, which helps to avoid overfitting by shrinking less important coefficients to zero. The use of a nomogram, which provides a user-friendly, graphical representation of the prognostic model, making it easier for clinicians to apply in practice. Moreover, we undertook both internal and external validation procedures, a preemptive measure to avert overfitting, to ensure the robustness of the model. Based on the model, it is possible to predict the probability and risk of storage-related lesions in platelets over periods of 3-, 5-, and 7-days under various storage conditions.

Our current study is limited because it has a small sample size, with limited generalizability as all samples are from China, and the distribution of the enrolled characteristics might be different in other areas and storage strategies, making it susceptible to the inherent biases of such a study format. Hence, our results should be further improved by prospective studies in multicenter and large-scale trials and future research should focus on the recovery, life span, or biological function of platelets *in vivo* predicted by this model.

In summary, based on miRNA sequencing technology, this study identified 13 differentially expressed miRNAs associated with platelet storage lesions. By utilizing the LASSO-Cox regression model, a nomogram-based classifier was established to effectively predict the storage lifespan of platelets and categorize PC into high-risk and low-risk of PSLs occurrence. By accurately predicting the development of platelet storage lesions under different storage conditions, our nomogram-based model has the potential to transform clinical practice. It could enable healthcare providers to make informed decisions about platelet inventory management, optimizing the use of resources and ensuring that patients receive the highest quality platelets. Additionally, the implications of our research are substantial, as it may lead to more efficient and effective platelet transfusion therapy, which is essential for the health and wellbeing of patients requiring these life-saving treatments.

## Data Availability

The datasets presented in this study can be found in online repositories. The names of the repository/repositories and accession number(s) can be found below: https://www.ncbi.nlm.nih.gov/geo/, GSE243571 https://zenodo.org/records/13910650, DOI: 10.5281/zenodo.13910649.

## Supplementary material

SUPPLEMENTARY DATA SHEET 1
The raw data of qRT-PCR.
