# Supplementary material for: Establishment of a nomogram-based prognostic model (LASSO-Cox regression) for predicting platelet storage lesions under different storage conditions
Source: Front Mol Biosci. 2025 Mar 31;12:1561114. doi: 10.3389/fmolb.2025.1561114 (PMC11994887; doi:10.3389/fmolb.2025.1561114)
Supplement: Supplementary file 1 [file Supplementaryfile1.pdf]

Establishment of a nomogram-based prognostic model (LASSO-Cox regression) for predicting platelet storage lesions under different storage conditions

Jun Xiao [1], Huimin Li [1], Xiaowei Li [1], Daoju Ren [2], Huifen Lei [1], Zhicai Li [2], Cuiying Li [1,2,*]

[1] Department of Blood Transfusion, Air Force Medical Center, Air force medical University, Beijing 100142, China
[2] The Fifth School of Clinical Medicine, Anhui Medical University, Hefei 230032, China
*** Correspondence:**
Cuiying Li
lcy2013@fmmu.edu.cn

Supplementary Table S1: The Cutoff Value of each miRNA expression based on X-tile

miRNAs Name	Cutoff Value (Fold Change)
hsa-miR-6891-5p	4.04
hsa-miR-4485-3p	3.01
hsa-miR-3138	2.37
hsa-miR-12136	2.75
hsa-miR-4485-5p	2.89
hsa-miR-25-5p	1.48
hsa-miR-652-5p	0.57
hsa-miR-148b-5p	0.41
hsa-miR-142-5p	0.47
hsa-miR-376a-5p	0.44
hsa-miR-378a-5p	0.46
hsa-miR-641	0.36
hsa-miR-101-3p	0.31

Supplementary Table S2 The target genes of differential expressed miRNAs

miRNA	Gene	miRDB	miRTarBase	TargetScan	Sum
hsa-miR-101-3p	CERS2	1	1	1	3
hsa-miR-101-3p	MYCN	1	1	1	3
hsa-miR-101-3p	RORA	1	1	1	3
hsa-miR-101-3p	ZNF654	1	1	1	3
hsa-miR-101-3p	FAM103A1	1	1	1	3
hsa-miR-101-3p	NUPL2	1	1	1	3
hsa-miR-101-3p	ANKRD17	1	1	1	3
hsa-miR-101-3p	NACA	1	1	1	3
hsa-miR-101-3p	LIN7C	1	1	1	3
hsa-miR-101-3p	ZEB1	1	1	1	3
hsa-miR-101-3p	OTUD4	1	1	1	3
hsa-miR-101-3p	TET2	1	1	1	3
hsa-miR-101-3p	CBFA2T2	1	1	1	3
hsa-miR-101-3p	FBXW7	1	1	1	3
hsa-miR-101-3p	MRPL42	1	1	1	3
hsa-miR-101-3p	SMARCD1	1	1	1	3
hsa-miR-101-3p	MTSS1L	1	1	1	3
hsa-miR-101-3p	TGFBR1	1	1	1	3
hsa-miR-101-3p	MFSD6	1	1	1	3
hsa-miR-101-3p	STAMBP	1	1	1	3
hsa-miR-101-3p	RAB39B	1	1	1	3
hsa-miR-101-3p	AEBP2	1	1	1	3
hsa-miR-101-3p	TMEM170B	1	1	1	3
hsa-miR-101-3p	KLF6	1	1	1	3
hsa-miR-101-3p	RAP2C	1	1	1	3
hsa-miR-101-3p	KIAA1462	1	1	1	3
hsa-miR-101-3p	ATXN1L	1	1	1	3
hsa-miR-101-3p	PTGS2	1	1	1	3
hsa-miR-101-3p	C1orf52	1	1	1	3
hsa-miR-101-3p	NEK7	1	1	1	3
hsa-miR-101-3p	TMED5	1	1	1	3
hsa-miR-101-3p	ICK	1	1	1	3
hsa-miR-101-3p	LMNB1	1	1	1	3
hsa-miR-101-3p	BCL9	1	1	1	3
hsa-miR-101-3p	FBN2	1	1	1	3
hsa-miR-101-3p	ARAP2	1	1	1	3
hsa-miR-101-3p	ARID1A	1	1	1	3

hsa-miR-101-3p	CAPN2	1	1	1	3
hsa-miR-101-3p	EED	1	1	1	3
hsa-miR-101-3p	KDM6B	1	1	1	3
hsa-miR-101-3p	EZH2	1	1	1	3
hsa-miR-101-3p	NACA2	1	1	1	3
hsa-miR-101-3p	INO80D	1	1	1	3
hsa-miR-101-3p	CADM1	1	1	1	3
hsa-miR-101-3p	N4BP1	1	1	1	3
hsa-miR-101-3p	MAML3	1	1	1	3
hsa-miR-101-3p	RANBP9	1	1	1	3
hsa-miR-101-3p	DDIT4	1	1	1	3
hsa-miR-101-3p	SACM1L	1	1	1	3
hsa-miR-101-3p	ABHD17C	1	1	1	3
hsa-miR-101-3p	FZD6	1	1	1	3
hsa-miR-101-3p	RAB5A	1	1	1	3
hsa-miR-101-3p	DUSP1	1	1	1	3
hsa-miR-101-3p	BICD2	1	1	1	3
hsa-miR-101-3p	CDH5	1	1	1	3
hsa-miR-101-3p	MLEC	1	1	1	3
hsa-miR-101-3p	MOB4	1	1	1	3
hsa-miR-101-3p	TNPO1	1	1	1	3
hsa-miR-101-3p	SUB1	1	1	1	3
hsa-miR-101-3p	TGFBR3	1	1	1	3
hsa-miR-101-3p	HSPE1-MOB4	1	1	1	3
hsa-miR-101-3p	PIP5K1C	1	1	1	3
hsa-miR-101-3p	RAC1	1	1	1	3
hsa-miR-101-3p	PRKAA1	1	1	1	3
hsa-miR-101-3p	MPPE1	1	1	1	3
hsa-miR-101-3p	CDKN1A	1	1	1	3
hsa-miR-101-3p	NLK	1	1	1	3
hsa-miR-101-3p	DNMT3A	1	1	1	3
hsa-miR-101-3p	FAR1	1	1	1	3
hsa-miR-101-3p	SGPL1	1	1	1	3
hsa-miR-101-3p	LRCH2	1	1	1	3
hsa-miR-101-3p	GNB1	1	1	1	3
hsa-miR-101-3p	ATXN1	1	1	1	3
hsa-miR-101-3p	MNX1	1	1	1	3
hsa-miR-101-3p	AP1S3	1	1	1	3
hsa-miR-101-3p	FOS	1	1	1	3
hsa-miR-101-3p	HNRNPF	1	1	1	3
hsa-miR-101-3p	TBC1D12	1	1	1	3
hsa-miR-101-3p	BCL2L11	1	1	1	3

hsa-miR-101-3p	UBN2	1	1	1	3
hsa-miR-101-3p	RAP1B	1	1	1	3
hsa-miR-101-3p	LCOR	1	1	1	3
hsa-miR-101-3p	KDM3B	1	1	1	3
hsa-miR-101-3p	SPATA2	1	1	1	3
hsa-miR-101-3p	ZNF207	1	1	1	3
hsa-miR-101-3p	UBE2A	1	1	1	3
hsa-miR-101-3p	ZBTB21	1	1	1	3
hsa-miR-101-3p	STMN1	1	1	1	3
hsa-miR-101-3p	SIX4	1	1	1	3
hsa-miR-101-3p	MBNL1	1	1	1	3
hsa-miR-101-3p	ANKRD11	1	1	1	3
hsa-miR-101-3p	SLC38A2	1	1	1	3
hsa-miR-101-3p	MRGBP	1	1	1	3
hsa-miR-101-3p	11-Sep	1	1	1	3
hsa-miR-101-3p	SLC39A6	1	1	1	3
hsa-miR-101-3p	ZCCHC2	1	1	1	3
hsa-miR-101-3p	PIK3C2B	1	1	1	3
hsa-miR-101-3p	RNF219	1	1	1	3
hsa-miR-101-3p	ZFP36L2	1	1	1	3
hsa-miR-101-3p	KIAA1586	1	1	1	3
hsa-miR-142-5p	FAM107B	1	1	1	3
hsa-miR-142-5p	SLC38A2	1	1	1	3
hsa-miR-142-5p	FEM1C	1	1	1	3
hsa-miR-142-5p	RAB10	1	1	1	3
hsa-miR-142-5p	PCBP1	1	1	1	3
hsa-miR-142-5p	NFE2L2	1	1	1	3
hsa-miR-142-5p	ERH	1	1	1	3
hsa-miR-142-5p	LUZP2	1	1	1	3
hsa-miR-142-5p	PTP4A1	1	1	1	3
hsa-miR-142-5p	RGPD4	1	1	1	3
hsa-miR-142-5p	LEPROT	1	1	1	3
hsa-miR-142-5p	RPS6KA5	1	1	1	3
hsa-miR-142-5p	ZFYVE21	1	1	1	3
hsa-miR-142-5p	SON	1	1	1	3
hsa-miR-142-5p	REST	1	1	1	3
hsa-miR-142-5p	SLC24A2	1	1	1	3
hsa-miR-142-5p	CEP97	1	1	1	3
hsa-miR-142-5p	ZFYVE26	1	1	1	3
hsa-miR-142-5p	TRAPPC2	1	1	1	3
hsa-miR-142-5p	EGLN3	1	1	1	3
hsa-miR-142-5p	RHOC	1	1	1	3
hsa-miR-142-5p	CAPRIN2	1	1	1	3

hsa-miR-142-5p	CREBRF	1	1	1	3
hsa-miR-142-5p	EFCAB14	1	1	1	3
hsa-miR-142-5p	PTPN4	1	1	1	3
hsa-miR-142-5p	DUSP2	1	1	1	3
hsa-miR-142-5p	FIGN	1	1	1	3
hsa-miR-142-5p	SLC30A7	1	1	1	3
hsa-miR-142-5p	BRIX1	1	1	1	3
hsa-miR-142-5p	ULK1	1	1	1	3
hsa-miR-142-5p	ROBO1	1	1	1	3
hsa-miR-142-5p	ACTN4	1	1	1	3
hsa-miR-142-5p	FGFR1OP	1	1	1	3
hsa-miR-142-5p	TGFBR2	1	1	1	3
hsa-miR-142-5p	ZBTB43	1	1	1	3
hsa-miR-142-5p	MED17	1	1	1	3
hsa-miR-142-5p	PCBP2	1	1	1	3
hsa-miR-148b-5p	NSD1	1	1	1	3
hsa-miR-25-5p	MICAL2	1	1	1	3
hsa-miR-3138	RPL10	1	1	1	3
hsa-miR-3138	SDHD	1	1	1	3
hsa-miR-376a-5p	BZW1	1	1	1	3
hsa-miR-376a-5p	PTPRJ	1	1	1	3
hsa-miR-376a-5p	RAP2A	1	1	1	3
hsa-miR-376a-5p	VMA21	1	1	1	3
hsa-miR-376a-5p	PTPN4	1	1	1	3
hsa-miR-376a-5p	DAB2	1	1	1	3
hsa-miR-378a-5p	UBN2	1	1	1	3
hsa-miR-378a-5p	TMOD2	1	1	1	3
hsa-miR-378a-5p	YEATS2	1	1	1	3
hsa-miR-378a-5p	CYLD	1	1	1	3
hsa-miR-378a-5p	SUFU	1	1	1	3

hsa-miR-378a-5p	GABPA	1	1	1	3
hsa-miR-378a-5p	STAC2	1	1	1	3
hsa-miR-378a-5p	VAMP4	1	1	1	3
hsa-miR-378a-5p	ABHD15	1	1	1	3
hsa-miR-378a-5p	TUSC2	1	1	1	3
hsa-miR-378a-5p	EIF2B2	1	1	1	3
hsa-miR-378a-5p	CXorf23	1	1	1	3
hsa-miR-378a-5p	SGTB	1	1	1	3
hsa-miR-4485-5p	HM13	1	1	1	3
hsa-miR-4485-5p	SETD1B	1	1	1	3
hsa-miR-4485-5p	CDK9	1	1	1	3
hsa-miR-4485-5p	MCM4	1	1	1	3
hsa-miR-641	DDX3X	1	1	1	3
hsa-miR-641	MAP1B	1	1	1	3
hsa-miR-641	SLC35G2	1	1	1	3
hsa-miR-641	KPNA6	1	1	1	3
hsa-miR-641	UBE2V2	1	1	1	3
hsa-miR-641	NUFIP2	1	1	1	3
hsa-miR-641	CLDND1	1	1	1	3
hsa-miR-641	SDE2	1	1	1	3
hsa-miR-641	MAPK10	1	1	1	3
hsa-miR-641	CREBRF	1	1	1	3
hsa-miR-641	EIF4A2	1	1	1	3
hsa-miR-641	DEPDC1B	1	1	1	3
hsa-miR-641	KIF5B	1	1	1	3
hsa-miR-641	ZNF106	1	1	1	3
hsa-miR-641	NRIP1	1	1	1	3
hsa-miR-641	STAM	1	1	1	3
hsa-miR-641	KLHL32	1	1	1	3
hsa-miR-641	THSD7A	1	1	1	3
hsa-miR-652-5p	ATF7IP	1	1	1	3

hsa-miR-6891-5p	SSPN	1	1	1	3
hsa-miR-6891-5p	HMGXB3	1	1	1	3
hsa-miR-6891-5p	YWHAE	1	1	1	3
hsa-miR-6891-5p	DDX11	1	1	1	3
hsa-miR-6891-5p	KLHDC3	1	1	1	3
hsa-miR-6891-5p	SET	1	1	1	3
hsa-miR-6891-5p	ARID1A	1	1	1	3
hsa-miR-6891-5p	IGF2	1	1	1	3
hsa-miR-6891-5p	FAM168A	1	1	1	3
hsa-miR-6891-5p	CHRDL1	1	1	1	3
hsa-miR-6891-5p	BSDC1	1	1	1	3
hsa-miR-6891-5p	MRRF	1	1	1	3
hsa-miR-6891-5p	PPP1R1C	1	1	1	3
hsa-miR-6891-5p	EN2	1	1	1	3
hsa-miR-6891-5p	PLA2G2F	1	1	1	3
hsa-miR-6891-5p	NUFIP2	1	1	1	3
hsa-miR-6891-5p	TOB2	1	1	1	3
hsa-miR-6891-5p	TEX261	1	1	1	3
hsa-miR-6891-5p	EIF4EBP1	1	1	1	3
hsa-miR-6891-5p	SDK1	1	1	1	3
hsa-miR-6891-5p	B4GALT1	1	1	1	3
hsa-miR-6891-5p	CHD4	1	1	1	3

hsa-miR-6891-5p	BTG2	1	1	1	3
hsa-miR-6891-5p	SCD	1	1	1	3
hsa-miR-6891-5p	MECP2	1	1	1	3

Supplementary Table S3 Baseline data of general information in the training and validation datasets

Variables	Training dataset (n=174)	Internal Validation dataset (n=75)	External Validation dataset (n=71)
SolubleCD40L (median (IQR))	1227.0(1072.0,1332.0)	1202.0(1080.0,1324.0)	1221.0(1035.0,1376.0)
TNF-α (median (IQR))	205.0(194.0,214.0)	204.0(192.0,211.0)	202.0(190.0,215.0)
Aggregation ratio (median (IQR))	66.0(58.0,73.0)	67.0(58.5,73.0)	66.0(59.0,72.0)
CD62P (median (IQR))	34.1(31.0,37.3)	34.1(31.6,37.5)	34.0(31.3,36.4)
has-miR-6891-5p			
Low	149(85.6%)	62(82.7%)	56(78.9%)
High	25(14.4%)	13(17.3%)	15(21.1%)
has-miR-4485-3p			
Low	148(85.1%)	63(84.0%)	56(78.9%)
High	26(14.9%)	12(16.0%)	15(21.1%)
has-miR-3138			
Low	133(76.4%)	53(70.7%)	50(70.4%)
High	41(23.6%)	22(29.3%)	21(29.6%)
has-miR-12136			
Low	146(83.9%)	65(86.7%)	60(84.5%)
High	28(16.1%)	10(13.3%)	11(15.5%)
has-miR-4485-5p			
Low	143(82.2%)	58(77.3%)	59(83.1%)
High	31(17.8%)	17(22.7%)	12(16.9%)
has-miR-25-5p			
Low	127(73.0%)	56(74.7%)	50(70.4%)
High	47(27.0%)	19(25.3%)	21(29.6%)
has-miR-652-5p			
Low	48(27.6%)	22(29.3%)	22(31.0%)
High	126(72.4%)	53(70.7%)	49(69.0%)
has-miR-148b-5p			
Low	55(31.6%)	27(36.0%)	22(31.0%)
High	119(68.4%)	48(64.0%)	49(69.0%)
has-miR-142-5p			
Low	53(30.5%)	19(25.3%)	23(32.4%)
High	121(69.5%)	56(74.7%)	48(67.6%)
has-miR-376a-5p			
Low	71(40.8%)	36(48.0%)	30(42.3%)
High	103(59.2%)	39(52.0%)	41(57.7%)
has-miR-378a-5p			
Low	80(46.0%)	32(42.7%)	33(46.5%)
High	94(54.0%)	43(57.3%)	38(53.5%)
has-miR-641			
Low	59(33.9%)	33(44.0%)	24(33.8%)
High	115(66.1%)	42(56.0%)	47(66.2%)
has-miR-101-3p			
Low	47(27.0%)	21(28.0%)	18(25.4%)
High	127(73.0%)	54(72.0%)	53(74.6%)
Storage method			
22℃	74(42.5%)	28(37.3%)	24(33.8%)
4℃	71(40.8%)	26(34.7%)	24(33.8%)
RT transport	29(16.7%)	21(28.0%)	23(32.4%)

RT: Room Temperature

Supplementary Table S4 Univariate and multivariate Cox hazards regression analysis of factors associated with PSLs in the training dataset

Variables	Univariate analysis			Multivariate analysis		
	HR	95% CI	*P* value	HR	95% CI	*P* value
Storage method	0.999	0.682-1.464	0.997	1.295	0.826-2.032	0.260
has-miR-101-3p	0.325	0.186-0.566	<0.001			
hsa-miR-641	0.208	0.116-0.375	<0.001			
hsa-miR-378a-5p	0.167	0.083-0.334	<0.001			
hsa-miR-376a-5p	0.201	0.107-0.379	<0.001			
hsa-miR-142-5p	0.281	0.161-0.492	<0.001			
hsa-miR-148b-5p	0.084	0.043-0.166	<0.001	0.171	0.078-0.377	<0.001
hsa-miR-652-5p	0.159	0.089-0.282	<0.001			
hsa-miR-25-5p	6.838	3.821-12.236	<0.001	2.058	0.921-4.600	0.079
hsa-miR-4485-5p	5.084	2.894-8.931	<0.001			
hsa-miR-12136	7.154	4.074-12.561	<0.001	1.354	0.659-2.782	0.410
hsa-miR-3138	5.925	3.36-10.448	<0.001			
hsa-miR-4485-3p	6.688	3.774-11.85	<0.001	2.280	1.212-4.288	0.011
hsa-miR-6891-5p	5.600	3.162-9.918	<0.001			

Supplementary figures legends

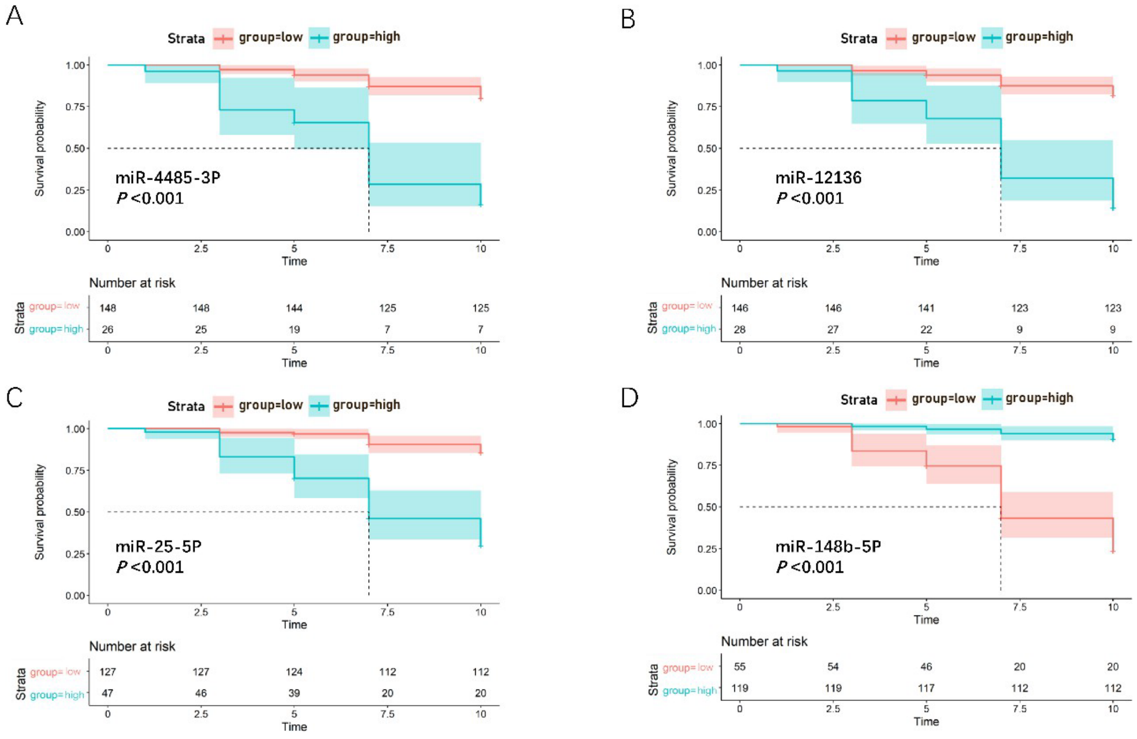

Supplementary Figure S1: The Kaplan-Meier curve analysis of selected miRNAs. The Kaplan-Meier curves of miR-4485-3p (A), miR-12136 (B), miR-25-5p (C), and miR-148b-5p (D) were plotted in the training dataset.

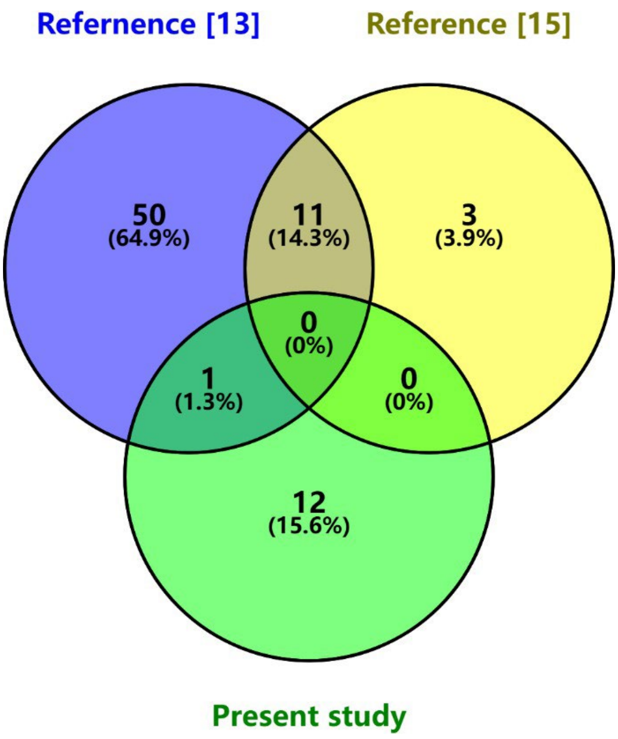

Supplementary Figure S2: The Venn diagram of differential miRNAs in different test sets.
